# The effectiveness of individual placement and support for people with mental illness new on social benefits: a study protocol

**DOI:** 10.1186/1471-244X-13-195

**Published:** 2013-07-24

**Authors:** Sandra Viering, Bettina Bärtsch, Caitriona Obermann, Nicolas Rüsch, Wulf Rössler, Wolfram Kawohl

**Affiliations:** 1 Department of Psychiatry, Psychotherapy and Psychosomatics, University Hospital for Psychiatry, Militärstrasse 8, 8021, Zurich, Switzerland; 2 University of Zurich, Militärstrasse 8, 8021, Zurich, Switzerland

## Abstract

**Background:**

In Switzerland, people with a severe mental illness and unable to work receive disability benefits (‘IV-pension’). Once they are granted these benefits, the chances to regain competitive employment are usually small. However, previous studies have shown that individual placement and support (IPS) supports a successful reintegration into competitive employment. This study focuses on the integration of newly appointed IV-pensioners, who have received an IV-pension for less than a year.

**Method/design:**

The present pilot project ZHEPP (Zürcher Eingliederungs-Pilot Projekt; engl.: Zurich integration pilot project) is a randomized controlled trial (RCT). The 250 participants will be randomized to either the intervention or the control group. The intervention group receives support of a job coach according to the approach of IPS. Participants in the control group do not receive IPS support. Participation takes a total of two years for each participant. Each group is interviewed every six months (T_0_-T_4_). A two-factor analysis of variance will be conducted with the two factors *group* (intervention versus control group) and *outcome* (employment yes/no). The main criterion of the two-factor analysis will be the number of competitive employment contracts in each group.

**Discussion:**

This study will focus on the impact of IPS on new IV-pensioners and aims to identify predictors for a successful integration. Furthermore, we will examine the effect of IPS on stigma variables and recovery orientation.

**Trial registration:**

ISRCTN54951166

## Background

In general only 10 to 20% of people who suffer from severe mental illness are employed in the competitive employment market [1]. However, most people suffering from a severe mental illness would actually like to work in the competitive employment market [2]. As persons with mental illness often perceived as unreliable employees by potential employers, the reintegration can be very difficult [3]. Additionally, health professionals frequently discourage patients from applying for competitive employment, because they are convinced that a stressful surrounding will lead to a destabilization of the patient [4,5].

In Switzerland, people suffering from a physical or mental illness which prevents them from working receive a financial support (so called IV-pension) from the social services SVA Zurich (Sozialversicherungsanstalt Zürich, engl.: social benefits centre of Zurich, so called IV-institution). Over the past 12 years the IV-institution has registered a continual increase of people receiving an IV-pension because of mental illness. In the year 2000 around 77 000 people received this contribution in Switzerland but by 2011 the amount of IV-pensioners had increased to 102 000.

Before receiving the IV-pension, the IV-institution offers several possibilities of supporting a reintegration into the competitive employment market (e.g. vocational training, intervention of apprenticeship). However, after receiving the approval for an IV-pension auxiliary measures to support a reintegration are limited. In consequence IV-pensioners may not feel sufficiently supported by the IV-insurance. During our information gatherings many IV-pensioners stated that the only thing they knew about the IV-insurance was that it provided assistance in finding sheltered work.

Most mentally ill people including IV-pensioners state that they are not keen to work in such places and would rather be integrated into competitive employment [4]. They feared that working in a sheltered work place will lead to a high risk of having to stay in this environment [6].

Furthermore, patients state that the integration into the competitive employment market leads to more self-esteem and an increased quality of life [7] due to receiving a salary as well as the chance of finding more social contacts [8]. Mentioning working in a sheltered employment also means revealing one’s illness and hence a fear of not being accepted in society [9].

The stigma associated with psychiatric disorders can have a major negative impact on individuals with mental illness. Stigma comes in two forms [10]. First, members of the general public, for example employers, can endorse negative stereotypes about people with mental illness and discriminate against them, e.g. not inviting persons with mental illness for job interviews. This is referred to as public stigma. Second, self-stigma occurs if people with mental illness agree with those negative stereotypes and turn them against themselves, undermining their self-esteem, self-efficacy and motivation to pursue life goals such as employment [11]. People with mental illness can also suffer from stigma as a stressor if they perceive stigma as a potential harm that exceeds their coping resources [12,13].

There are two main reasons to examine stigma variables in the context of IPS [14]: (i) fear of public stigma as well as self-stigma may stop people from seeking competitive employment, therefore increased levels of stigma variables could predict less positive IPS outcomes; (ii) re-entering the workforce and the associated social roles and contacts might lead to an increased perception of social inclusion among people with mental illness, resulting in less self-stigma, perceived public stigma as well as stigma-related stress; therefore stigma variables can be assessed as secondary outcomes of IPS.

The traditional approach in rehabilitating people with mental illness has been *first train*, *then place*[15]. In this approach possible difficult situations concerning the employment are trained beforehand. After training these situations the person is placed in competitive employment. Practicing the various scenarios which could occur in the future employment is useful, but the situations in real life are generally much tougher than expected and differ from the trained situations. In many cases the job was chosen for the client, and not by the client, and was thus not suited to the client`s needs and preferences [1]. *First train*, *then place* has drawbacks, as people become ill and are not able to hold their jobs for a long time [16]. In consequence many of the people treated by this approach are only able to work in a sheltered workplace [17].

As frontline services to integrate IV-pensioners into the competitive employment market are almost non-existent, two problems emerge.

Firstly, IV-pensioners often lack the knowledge of the legal framework and are unsure about their rights. Erroneously, they commonly believe that they are not allowed to apply for a job in the competitive employment market while receiving an IV-pension. The IV-insurance is addressing this issue by providing more information to their pensioners. Secondly, pensioners grow accustomed to not working, thereby increasing the risk of further chronification. To minimize such risks this study includes only new IV-pensioners who have received an IV-pension for less than one year.

During the 1980s the concept of supported employment (SE) was developed in the USA [18] as a reaction to the inefficient *first train*, *then place*-models [19]. SE includes intensive job-search assistance for people with mental illness. Particular attention is given to the clients’ preferences and skills, to assist successful integration into the competitive employment market and to support them while working in competitive employment market [20]. In the USA this approach has been proven to be very successful [21]. However, a transfer of these methods to Europe requires certain structural changes, as Europe differs from the USA concerning job market and welfare system. In 2007 EQOLISE (Enhancing the Quality of Life and Independence of Persons Disabled by Severe Mental Illness through Supported Employment, [19]) was established. In this study, the first of its kind in Europe, participants were trained by job coaches according to the supported employment approach of Individual Placement and Support (IPS) [22]. This study was implemented in six centres across Europe. The results showed that participants assigned to the IPS group were employed more often into competitive employment as compared to the control group. In addition, the IPS participants dropped out of the study less often, and the rates of hospitalization were lower.

Moreover, EQOLISE demonstrated that working in a sheltered work place did not lead to a comparable stabilization of the affected persons [19]. A better state of mental health (e.g. lower anxiety and depression) and a more successful integration into the competitive employment market through the approach of IPS was evident [23].

The supported employment concept IPS, which was used in the EQOLISE study as well as in the study presented here, ensures the immediate support of a job coach and the direct integration into competitive employment. Job coaches support the client by searching for vacant jobs, assisting applications, as well as coaching the client in working situations [24]. If the client and employer approve, job coaches may also support the employer and the workmates.

### Research need

The EQOLISE study was able to prove that the positive effects for the participants assigned to the IPS group are comparable to findings in the US. However, previous studies did not differentiate between participants with different characteristics and thus no conclusions for subgroups of clients such as persons with new-onset disability benefits can be drawn. After receiving an IV-pension the risk of further chronification of a mental illness increases over time, hence making a successful reintegration into the competitive employment market increasingly difficult.

This study is a randomized controlled trial (RCT) and will investigate the effectiveness of SE for IV-pensioners receiving the IV-pension for less than a year.

### Research question

The main aim of this study is:

(1) To determine if job coaching according to the IPS approach leads to a more successful integration of new IV-pensioners into competitive employment.

In addition, the following issues will be analyzed:

(2) Possible enhancement of quality of life, state of mental health and level of self-esteem by finding competitive employment

(3) Chances of maintaining an employment and increasing the workload, if the participant desires this and has the ability

(4) Cost-benefit equation of job coaching and potential saving schemes as compared to the current handling

(5) Predictors for a successful coaching after receiving an IV-pension

(6) The effect of IPS on stigma variables (perceived public stigma, self-stigma, stigma stress) and recovery orientation

(7) Stigma variables and recovery orientation as predictors of finding competitive employment during IPS

## Methods/design

### Time scale

To determine interest in participating in the pilot study ZHEPP, the project has been divided into two phases. The first phase was implemented to ascertain whether enough IV-pensioners were interested in participating in this study. The first phase involved the recruitment of 40 participants and those assigned to the intervention group involved in job coaching. The number of participants was easily reached and the first phase was successfully completed by the summer of 2011. The second phase was then initiated. A further 210 participants had to be recruited to reach the final sample size of 250. This recruitment took place until September 2012. After being assigned to the control or intervention group the participants will be followed up over the next two years. Hence the total duration of the study will be three years and nine months (January 2011 until end of September 2014).

### Recruitment and design

Persons who recently received the approval of the IV-pension were invited to an information event. The aim was to inform about the project as well as the supported employment concept IPS. Additionally the job coaches were introduced to the potential participants.

Those interested in participating in our study had the possibility to register and were invited to an one-on-one dialog for further information. Persons who subsequently wished to participate in the study signed the informed consent and were randomized to one of the two groups (control group or the intervention group). During the following two years the participants will be interviewed every six months concerning their well-being, self-esteem, stigma and recovery variables as well as job status (T_0_-T_4_) (List of instruments see Table 1).

**Table 1:** Overview questionnaire based instruments

**Instrument**	**Variable**	**Perspective**	**Measurement point**				
			**T0**	**T1**	**T2**	**T3**	**T4**
Incentive focus scale	Motivation of participant	P	√	√	√	√	√
Client Sociodemographic and Service Receipt Inventory	Sociodemographic facts; medical supply	P/R	√	√	√	√	√
Lacanshire Quality of Life Profile	Quality of Life	P/R	√	√	√	√	√
Symptom checklist 90-revised	Psychological symptoms	P/R	√	√	√	√	√
Rosenberg self esteem scale	Self-esteem	P/R	√	√	√	√	√
Groningen Social Disability Scale	Social role; social disability	P/R	√	√	√	√	√
Global Assessment of Social Disability Scale	Overall psychological disturbance	R	√	√	√	√	√
Internalizes Stigma of Mental Illness Scale	Stigma coping; social drawback	P	√		√		√
Cognitive appraisal	Cognitive appraisal Stigmatization as stressor	P	√		√		√
Perceived Devaluation-Discrimination Questionnaire	Experienced Stigmatization	P	√		√		√
Recovery Assessment Scale	How they feel about themselves and their life	P	√		√		√
Centre für Epidemiological Studies Depression Scale	Depressive symptoms	P	√		√		√
Job discrimination	Experienced discrimination at working place	P	√		√		√
Client Job Status	Working situation	P/JC	√	√	√	√	√
Job Preferences	Job preferences	P/JC	√				
Indiana Job Satisfaction/ Termination Scale	Job satisfaction beginning, while and at the end	P/JC	√	√	√	√	√

*Note*: P: participant, R: Researcher, P/R: Patient by communicating with researcher, P/JC: Patient by communication with job coach; Measurement point: compiled every six month (T0, T1, T2, T3, T4).

The ZHEPP project is a randomized control trial (RCT) with two factors, *group* (intervention versus control group) and *outcome* (employment yes/no).

The participants of the intervention group are supported by a job coach according to the approach of IPS. Those randomized to the control group are not subject to any intervention by our job coaches, but are merely invited to an interview every six months. The catchment area is the canton of Zurich, Switzerland.

### Sample size

The target study population consists of 250 IV-pensioners, who recently (not longer than a year) received the approval of the IV. In total, 126 persons were assigned to the intervention group, where they receive the coaching according to the IPS approach. The other 124 were assigned to the control group.

The sample size was calculated through power analysis. A medium effect size (0.42 SD) should be detected with a power of 95% at a two tailed significance level of 0.05.

Ideally, participants assigned to the intervention group can find employment in the competitive employment market and may subsequently become independent of social benefits subsequently.

### Inclusion and exclusion criteria

Inclusion criteria:

● IV-pension due to a mental disorder (full or part time pension)

● The participant does not receive the IV-pension for longer than a year

● The IV-pensioner`s goal is to work in the competitive employment market

● Ability to give written informed consent

● Working age (18–60)

Exclusion criteria:

● Organic mental disorder (-10: F0)Diagnosed to ICD

● Mental retardation (Diagnosed to ICD-10: F7)

### Interview based instruments and data collection

Based on the EQOLISE study the following instruments (German version) are used to quantify the outcome of the general well-being of the IV-pensioners. The participants are interviewed every 6 months by research workers (T_0_-T_4_).

(1) Incentive Focus Scale (IF): assessment of the participant`s motivation [25]

(2) Client Sociodemographic and Service Receipt Inventory (CSSRI- EU): assessment of sociodemographic facts and the medical supply [26,27]

(3) Lancashire Quality of Life Profile (LQoLP): assessment of the participant`s quality of live [28]

(4) Symptom-Checklist 90-Revised (SCL-90-R): assessment of the participant`s psychological symptoms [29]

(5) Rosenberg self-esteem scale (RSES): assessment of the participant`s self-esteem [30]

(6) Groningen Social Disability Scale (GSDS-II): assessment of social disabilities, fulfil their social role as expected [31]

(7) Global Assessment of Functioning (GAF): brief assessment of social, occupational and psychological functioning of the participant [32]

(8) Recovery Assessment Scale (RAS), 24-item short version: assessment of participant`s feeling towards him/herself and his/her life [33]

(9) Center for Epidemiological Studies Depression Scale (CES-D), German 15-item version: assessment of depressive symptoms [34]

This study will also assess stigma and recovery variables, using the following validated self-report measures administered once a year (T_0_, T_2_ and T_4_):

(1) Internalized Stigma of Mental Illness Scale (ISMI), 29 items [35]

(2) Cognitive appraisal of stigma as a stressor, 8 items [12,13]

(3) Perceived Devaluation-Discrimination Questionnaire (PDDQ), 12 items, measuring perceived public stigma [36]

Additionally, for those who worked at least one day in the last year.

Experienced discrimination at work, 5 items (Rüsch et al., unpublished). Furthermore, job preferences as well as the participant`s expectation concerning the process of finding an employment will be evaluated in the first interview (T_0_). Every six months (T_0_-T_4_) the current situation of employment and, if applicable, the participant`s satisfaction with his/her employment are assessed.

(1) Client Job Status: statement of the ongoing working situation of the participant

(2) Job Preferences: assessment of the participant`s job preferences

(3) Indiana Job-Satisfaction/Termination Scale (IJSS/IJTS): assessment of the participant`s satisfaction with his/her employment at the beginning, while having a job and if applicable after termination of her/his contract of employment

Moreover, job coaches are required to complete the IPS-Fidelity scale [37] every three months to ensure that the standards of the IPS concept are fulfilled.

### Plan of analysis

This study aims to determine, whether job coaching according to IPS enables the successful integration of IV-pensioners into competitive employment.

The total N of this study is 250 participants. Through a Bernoulli randomization the participants are assigned to either the control or the intervention group.

The intervention’s effectiveness is tested by interview based instruments (in total 16 questionnaires). Binary, nominal and interval-scaled data are collected.

A two-factor analysis of variance with the two factors *group* (intervention versus control group) and *outcome* (employment yes/no) will be conducted to control the effectiveness of the intervention. The main criterion of the two-factor analysis will be the quantity of jobs received.

As parameters vary on more than one level a simultaneous comparison in a multilevel model is carried out.

Moreover, the study contains two group comparisons. The persistence of being employed or not in both groups is examined with help of a Cox-regression.

A significance level < 0.05, thus, a confidence interval of 95% is to be achieved.

### Ethics

The study was approved by the ethics committee of the canton of Zurich (KEK) und the reference number KEK-ZH-NR: 2010-0311/0. Research is carried out in compliance with the Declaration of Helsinki of the World Medical Association (WMA).

## Discussion

Whilst there has been research about specific age groups [16], the effect of IPS on several other groups (e.g. new recipients of disability benefits) so far has not been demonstrated. Therefore, this study will focus on the impact of IPS on newly appointed IV-pensioners. Another aim will be to identify predictors for the coaching success in IPS and the relationship between self-stigma and coaching success.

ZHEPP is a randomized controlled trial (RCT). The participants of the study are randomized to either the control group or the intervention group. We hypothesize that job coaching according to IPS will lead to a more successful integration of new IV-pensioners into competitive employment. If our hypothesis is supported, IPS may in the future be offered to new Swiss IV-pensioners on a routine basis.

## Abbreviations

ZHEPP: Zürcher Eingliederungs Pilot Projekt engl.: Zurich integration pilot project; EQOLISE: Enhancing the Quality of Life and Independence of Persons Disabled by Severe Mental Illness through Supported Employment; IV-pension: Pension for disabled person; SVA: Sozialversicherungsanstalt social contribution centre of Zurich; SE: Supported Employment; IPS: Individual Placement and Support; RCT: Randomized control trial; KEK: Ethics committee of canton Zurich.

## Competing interests

The authors declare that they have no competing interests.

## Authors’ contributions

WK, BB, NR, and WR were responsible for the conception of the project and drafting the first study proposal. SV wrote the first draft and SV, WK and CO were involved in writing the manuscript. All authors critically revised and approved the final manuscript.

## Pre-publication history

The pre-publication history for this paper can be accessed here:

http://www.biomedcentral.com/1471-244X/13/195/prepub

## References

[B1] McGurk SR Mueser MT Pascaris A Cognitive training and supported employment of persons with severe mental illness: one- year results from s randomized controlled trial Schizophr Bull 2005 31 898 909 10.1093/schbul/sbi037 16079391

[B2] Bond GR Supported employment: evidence for an evidence-based practice Psychiatr Rehabil J 2004 27 345 359 15222147 10.2975/27.2004.345.359

[B3] Scheid TL Stigma as a barrier to employment: Mental disability and the Americans with Disabilities Act Int J Law Psychiatry 2005 28 670 690 10.1016/j.ijlp.2005.04.003 16112732

[B4] Drake RE McHugo GJ Bebout RR Becker DR Harris M Bond GR Quimby E A randomized clinical trial of supported employment for inner-city patients with severe mental disorders Arch Gen Psychiatry 1999 56 627 633 10.1001/archpsyc.56.7.627 10401508

[B5] Rinaldi M Perkins R Glynn E Montibeller T Clenaghan M Rutherford J Individual placement and support: from research to practice Adv Psychiatr Treat 2008 13 50 60

[B6] Bond GR Principles of the individual placement and support: empirical support Psychiatr Rehabil J 1998 22 11 23

[B7] Drake RE McHugo GJ Becker DR Anthony WA Clark RE The New Hampshire study of supported employment for people with severe mental illness Int J Psychosoc Rehabil 1996 64 391 399 10.1037//0022-006x.64.2.391 8871423

[B8] Bell MD Lysaker PH Milstein RM Clinical benefits of paid work activity in schizophrenia Schizophr Bull 1996 22 51 67 10.1093/schbul/22.1.51 8685664

[B9] Bond GR Becker DR Drake RE Rapp CA Meisler N Lehmann AF Bell MD Blyler CR Implementing supported employment as an evidence-based practice Psychiatr Serv 2001 52 313 322 10.1176/appi.ps.52.3.313 11239097

[B10] Rüsch N Angermeyer MC Corrigan PW Mental illness stigma: concepts, consequences, and initiatives to reduce stigma Eur Psychiatry 2005 20 529 539 10.1016/j.eurpsy.2005.04.004 16171984

[B11] Corrigan PW Larson JE Rüsch N Self-stigma and the "why try" effect: Impact on life goals and evidence-based practices World Psychiatry 2009 8 75 81 19516923 10.1002/j.2051-5545.2009.tb00218.x PMC2694098

[B12] Rüsch N Corrigan PW Powell K Rajah A Olschewski M Wilkniss S Batia K A stress-coping model of mental illness stigma: II. Emotional stress responses, coping behavior and outcome Schizophr Res2009a 110 65 71 10.1016/j.schres.2009.01.005 19237266 PMC2720565

[B13] Rüsch N Corrigan PW Wassel A Michaels P Olschewski M Wilkniss S Batia K A stress-coping model of mental illness stigma: I. Predictors of cognitive stress appraisal Schizophr Res2009b 110 59 64 10.1016/j.schres.2009.01.006 19269140 PMC2720567

[B14] Corrigan PW Powell KJ Rüsch N How does stigma affect work in people with serious mental illnesses? Psychiatr Rehabil J 2012 35 381 384 23116379 10.1037/h0094497

[B15] Matschnig T Frottier P Seyringer ME Frühwald S Arbeitsrehabilitation psychics kranker Menschen - ein Überblick über Erfolgsprädiktoren Psychiatr Prax 2008 35 271 278 10.1055/s-2007-986351 18504685

[B16] Twamley EW Narvaez JM Becker DR Bartels SJ Jeste DV Supported employment for middle-aged and older people with schizophrenia Psychiatr Rehabil J 2008 11 76 89 10.1080/15487760701853326 PMC2638571 19212460

[B17] Bond GR McDonel E Vocational rehabilitation outcomes for persons with psychiatric disabilities: an update Journal of Vocational Rehabilitation 1991 1 9 20

[B18] Bond GR Drake RE Mueser KT Becker DR An update on supported employment for people with severe mental illness Psychiatr Serv 1997 48 335 346 9057235 10.1176/ps.48.3.335

[B19] Burns T Catty J Becker T Drake RE Fioritti A Knapp M Lauber C Rössler W Tomov T Van Busschbach J White S Wiersma D on behalf of the EQOLISE The effectiveness of supported employment for people with severe mental illness: a randomised controlled trial Lancet 2007 370 1146 1152 10.1016/S0140-6736(07)61516-5 17905167

[B20] Campbell K Bond GR Drake RE Who benefits from supported employment: a meta-analytic study Schizophr Bull 2011 37 370 380 10.1093/schbul/sbp066 19661196 PMC3044633

[B21] Cook JA Leff HS Blyler CR Results of a multisite randomized trial of supported employment intervention for individuals with severe mental illness Arch Gen Psychiatry 2005 62 505 512 10.1001/archpsyc.62.5.505 15867103

[B22] Becker DR Drake RE Individual placement and support: a community mental health centre approach to vocational rehabilitation Community Ment Health J 1994 30 193 206 10.1007/BF02188630 8013215

[B23] Bond GR Drake RE Becker DR Generalizability of the individual placement and support (IPS) model of supported employment outside the US World Psychiatry 2012 11 32 39 10.1016/j.wpsyc.2012.01.005 22295007 PMC3266767

[B24] Burns T Catty J White S Becker T Koletsi M Fioritti A Rössler W Tomov T Van Busschbach J Wiersma D Lauber C The impact of supported employment and working on clinical and social functioning: results of an international study of individual placement and support Schizophr Bull 2009 35 949 958 10.1093/schbul/sbn024 18403375 PMC2728809

[B25] Rheinberg F Zweck und Tätigkeit 1989 Göttingen: Hogrefe

[B26] Chisholm D Client socio-demographic and service receipt inventory-European version: development of an instrument for international research: EPSILON study 5 Br J Psychiatry 2000 177 28 33 10.1192/bjp.177.39.s28 10945075

[B27] Roick C Kilian R Matschinger H Die deutsche version des client sociodemographic and service receipt inventory – Ein instrument zur erfassung psychiatrischer versorgungskosten Psychiatr Prax 2001 28 84 90 11605129 10.1055/s-2001-17790

[B28] Oliver JP Huxley PJ Priebe S Kaiser W Measuring the quality of life of severely mentally ill people using the Lancashire quality of life profile Soc Psychiatry Psychiatr Epidemiol 1997 32 76 83 10.1007/BF00788924 9050348

[B29] Derogatis LR SCL-90-R, administration, scoring, and procedures manual for the R(evised) version 1977 School of Medicine: Johns Hopkins University

[B30] Rosenberg M Society and the adolescent self-image Princeton 1965 Princeton: University Press

[B31] Wiersma D DeJong A Ormel J The Groningen Social Disability Schedule: “Development, relationship with I.C.I.D.H., and psychometric properties” Int J Rehabil Res 1988 11 213 224 10.1097/00004356-198809000-00001 2978514

[B32] Hall RC Global assessment of functioning. A modified scale Psychosomatics 1995 36 267 275 10.1016/S0033-3182(95)71666-8 7638314

[B33] Corrigan PW Salzer M Ralph RO Sangster Y Keck L Examining the factor structure of the recovery assessment scale Schizophr Bull 2004 30 1035 1041 10.1093/oxfordjournals.schbul.a007118 15957202

[B34] Schein RL Koenig HG The centre for epidemiological studies-depression (CES-D) scale: assessment of depression in the medically ill elderly Int J Geriatr Psychiatry 1997 12 436 446 10.1002/(SICI)1099-1166(199704)12:4<436::AID-GPS499>3.0.CO;2-M 9178047

[B35] Ritsher JB Otilingam PG Grajales M Internalized stigma of mental illness: psychometric properties of a new measure Psychiatry Res 2003 121 31 49 10.1016/j.psychres.2003.08.008 14572622

[B36] Link BG Understanding labeling effects in the area of mental disorders: an assessment of the effect of expectations of rejection Am J Community Psychol 1987 11 261 2736881109

[B37] Bond GR Vogler KM Resnick SG Evans LJ Drake RE Becker DR Dimensions of supported employment: factor structure of the IPS fidelity scale: work and mental health J Ment Health 2001 10 383 393

